# Src Family Kinases Inhibition Ameliorates Hypoxic-Ischemic Brain Injury in Immature Rats

**DOI:** 10.3389/fncel.2021.746130

**Published:** 2021-12-21

**Authors:** Han Qiu, Tianyang Qian, Tong Wu, Ting Gao, Qinghe Xing, Laishuan Wang

**Affiliations:** ^1^National Health Commission Key Laboratory of Neonatal Diseases, Department of Neonatology, Children's Hospital of Fudan University, Shanghai, China; ^2^Department of Neonatology, Children's Hospital of Fudan University and Institutes of Biomedical Sciences, Fudan University, Shanghai, China

**Keywords:** hypoxia-ischemia, Src family kinases, NMDA receptor, PP2, brain injury, behavioral test

## Abstract

Hypoxic-ischemic (HI) injury is one of the initial factors contributing to neonatal brain injury. Src family kinases (SFKs) are considered to act as molecular hubs for *N*-methyl-d-aspartate receptor (NMDAR) regulation and participate in the HI injury process. The objectives of this study were to evaluate the levels of phospho-Src (p-Src), the relationship between NMDARs and SFKs, and the effects of SFK inhibition on an immature rat HI brain injury model. The model was induced in 3-day-old Sprague–Dawley rats using the Rice-Vannucci model operation. The level of p-Src was evaluated using Western blotting. The association of NMDARs with SFKs was detected using Western blotting and coimmunoprecipitation. After intraperitoneal injection of 4-amino-5-(4-chlorophenyl)-7-(*t*-butyl) pyrazolo [3,4-*d*] pyrimidine (PP2), an SFK-selective inhibitor, neuropathological changes were observed by performing H&E and immunofluorescence staining, and the neurological functions were assessed using the following behavioral tests: modified neurological severity score, open field test, and Morris water maze test. The levels of p-Src first decreased at 0 h after injury, increased at 2 h after injury, and continuously decreased from 6 h to 3 days. Along with the increased p-Src levels observed at 2 h after injury, the phosphorylation of NMDAR subunit NR2B at tyrosine 1472 was increased. Following the administration of PP2, the increased p-Src and NMDAR-2B levels detected at 2 h after injury were decreased, and tissue injury and myelin basic protein expression were improved at 7 days after injury. The PP2 intervention improved the performance of injured rats on behavioral tests. In conclusion, we determined the patterns of p-Src expression after HI brain injury in immature rats and showed a relationship with the activated NMDA receptor. The inhibition of p-Src ameliorates neuropathological changes and damages neurological functions induced by HI injury.

## Introduction

A high risk of brain injuries has been observed in preterm survivors, resulting in cognitive, attentional, and motor deficits in 25–50% of patients (Back, 2017). The etiology of preterm brain injury is complex and includes the immature brain, the external insults, and the vulnerability of principal components, such as preoligodendrocytes (pre-OLs), cerebral cortex, subplate neurons, thalamus, and basal ganglia (Volpe, 2019). In addition, the injured brain shows a decrease in the number of subplate neuronal axons in the cortex and alterations in the maturation and myelination of regenerative OLs, decreasing the number of ensheathed axons (Volpe, 2009, 2019).

Src family kinases (SFKs) are non-receptor-type tyrosine kinases and include two subfamilies: Src- and Lyn-related kinases (Truong and Carroll, 2013). Three types of Src-related kinases, namely, Src, Fyn, and Yes, are expressed in the developing brain, which play important roles in many signaling pathways and various biological activities, such as gene transcription, cell adhesion, cell cycle progression, immune response, apoptosis, migration, and transformation (Stein et al., 1994; Mukherjee et al., 2020). Based on accumulating evidence, SFKs are important for neuronal outgrowth and myelination on OL (Umemori et al., 1994; Liang et al., 2004; Bauer et al., 2009; Rouer, 2010). One study was designed to administer SFK inhibitors to these neurons and showed that the physiological activity of SFKs was critical for neuronal survival (Iqbal Hossain et al., 2015). Brain-derived neurotrophic factor (BDNF) modulates the myelination of OLs by regulating the phosphorylation of SFKs through extracellular signal-regulated kinase (ERK) and receptors expressed on OLs (Peckham et al., 2016).

However, SFKs have important roles in regulating the function of *N*-methyl-d-aspartate receptors (NMDARs) (Salter and Kalia, 2004). Evidence shows that SFKs exert a regulatory effect upstream of NMDAR function to modulate synaptic plasticity and metaplasticity (Rajani et al., 2021). In an adult rat stroke model, the levels of activated NMDARs were increased after ischemia, which were related to Src and Fyn (Takagi et al., 1997; Cheung et al., 2000). In a neonatal mouse model of hypoxia-ischemia (HI) on a postnatal day 7, SFKs may regulate NMDARs and mediate downstream events (such as excitotoxicity) (Jiang et al., 2008). Changes in the levels of activated SFKs in the HI-injured immature brain remain unknown.

An SFK inhibitor, (4-amino-5-(4-chlorophenyl)-7-(*t*-butyl) pyrazolo [3,4-*d*] pyrimidine) (PP2), potently inhibits Lck and Src (Bain et al., 2007). Many studies have focused on PP2 to evaluate its neuroprotective effect. Intraperitoneal administration of PP2 after injury exerted some neuroprotective effects, and the dose of PP2 was approximately 1 μg/g (Jiang et al., 2008; Liu et al., 2010; Bai et al., 2014; Wu et al., 2015).

In this study, we identified the pattern of activated SFK expression in an immature rat HI brain injury model and analyzed the relationship of SFKs with NMDARs. Then, we detected the potential neuroprotective effects on HI injury by inhibiting activated SFKs with the SFK inhibitor, namely, PP2.

## Materials and Methods

### Animals

All animal studies were approved by the Ethics Committee of the Children's Hospital of Fudan University and were performed according to the National Institutes of Health guidelines. We chose Sprague–Dawley (SD) rats (Animal Science Laboratory of Fudan University) for our immature rat HI brain injury model. Both sexes of SD rats were used at P3, which corresponded to 23–32 weeks of human gestation (Mallard and Vexler, 2015).

### Immature Rat HI Brain Injury Model

Rats from the same litter were randomly divided into the control and HI groups. According to previous research (Huang et al., 2009), P3 rats in the HI group underwent right carotid artery ligation, followed by recovery and feeding with dams for 1.5 h. Then, the operated animals were placed in a chamber at 37°C and exposed to 6% oxygen for 2.5 h. Rats in the control group had neither right carotid artery ligation nor hypoxia.

We chose a subset of rats with HI to receive the SFK inhibitor, namely, PP2. PP2 (Tocris) was dissolved in 0.5% dimethyl sulfoxide (DMSO) followed by dilution (final DMSO concentration) in 0.9% normal saline. PP2 (1 μg/g, HI+PP2) or 0.5% DMSO in normal saline (vehicle control, HI+DMSO) was injected intraperitoneally at 0.5 h after hypoxia.

### Western Blotting

Equal amount of protein from the right hemisphere of the control or HI groups was separated on 4–12% SDS–PAGE gels (Beyotime) and transferred to polyvinyl difluoride membranes (Sigma Merck). The blots were then blocked with 5% bovine serum albumin in TBS buffer for 1 h at room temperature and probed overnight at 4°C by incubation with the following primary antibodies: Src (1:1,000, CST, catalog number: #2123), phosphor-Src (Tyr 416, 1:1,000, CST, catalog number: #6943), NMDAR subunit NR2B (1:1,000, CST, catalog number: #14544), phospho-NR2B (Tyr 1472, 1:1,000, CST, catalog number: #4208), myelin basic protein (MBP) (1:1,000, Biolegend, SMI99), Iba-1 (1:1,000, Abcam, ab178847), glial fibrillary acidic protein (GFAP) (1:1,000, CST, 12389), β-actin (1:5,000, Absin, abs137975), and GAPDH (1:1,000, Absin, abs132004), followed by appropriate secondary HRP-conjugated antibodies (anti-rabbit, 1:5,000, Absin, abs20002; anti-mouse, 1:5,000, Absin, abs20001). Blots were visualized with enhanced chemiluminescence reagents (BD Pharmingen) and Bio-Rad Image software.

### Immunoprecipitation

Equal amount of protein from the homogenized ipsilateral side (right hemisphere) of animals was incubated overnight at 4°C with 5 μl of the appropriate antibodies (1:40, rabbit anti-Src antibody, CST, catalog number: #2123), or normal rabbit IgG as a negative control. Subsequently, 20 μl of precleared magnetic beads (>400 μg IgG/ml, Bimark) were added to the protein sample to capture the immune complexes and incubated for 2 h at 4°C. After extensive washing, an equal volume of the eluate was separated on an 8% SDS–PAGE gel and transferred to a polyvinyl difluoride membrane. The blots were then reacted with NR2B (1:1,000, CST, catalog number: #14544) and Src (1:1,000, CST, catalog number: #2123) antibodies. Blots were visualized with enhanced chemiluminescence reagents (BD Pharmingen) and Bio-Rad Image software.

### H&E Staining, Nissl Staining, and Histological Evaluation

The SD rats aged between P3 and P17 were subjected to cardiac perfusion with normal cold saline and 4% paraformaldehyde (PFA). Brains were removed, fixed with 4% PFA overnight, and transferred to 20–30% sucrose solutions for dehydration. The brain was cut into coronal sections (10 μm) using a cryostat (Leica). After staining with an H&E kit and Nissl staining kit, images of sections were captured using a light microscope (Leica).

The volume of the brain sections and hippocampus was estimated using the following equation: *V* = *T* × area, where *V* is estimated volume, *T* is distance between the analyzed sections (10 μm), and area is the sum of points overlaid in the selected portion measured using ImageJ software.

### Immunofluorescence Staining

The sections were stained with anti-MBP (1:200, Biolegend, SMI99), anti-GFAP (1:200, CST, 12389), and anti-Iba-1 (1:100, Abcam, ab178847) antibodies at 4°C overnight, followed by incubation with appropriate fluorophore-conjugated secondary antibodies at room temperature (Cy3-conjugated goat anti-rabbit IgG antibody, 1:500, Jackson ImmunoResearch, 111-165-003; Cy3-conjugated donkey anti-mouse IgG antibody, 1:500, Jackson ImmunoResearch, 715-225-151). We used PBS to wash sections and Diamidinyl phenyl indole (DAPI) to detect cell nuclei. Images were captured using a fluorescence microscope (Leica).

### Behavioral Testing

All rats were placed in the testing room and familiarized with the surroundings before performing any behavioral tests. (1) At P17, we used the modified neurological severity score (mNSS) to detect the reflex, motor, sensory, and balance functions (Table 1) (Lin et al., 2020). (2) At P38, we performed the open field test to assess the distance traveled, time spent in exploring, and anxiety of rats. The rats were placed in the center of a transparent and open box (100 × 80 × 80 cm) located in the acoustic chamber. The motion trail was recorded within 5 min and analyzed using the JLBehv-LA program (Jiliang). (3) At P39-P43, the modified Morris water maze test was conducted to test the learning and spatial cognition abilities. We placed each rat in the pool at one of the four starting positions and allowed them to find the hidden platform under water each day for 4 days in the navigation trial. When rats located the platform or the swimming time was up to 2 min, the trial was finished, and the motion trail was recorded and analyzed using the JLBehv-M program (Jiliang) to measure the swimming time and distance. The spatial probe trial was performed on the 5th day (P43). The platform was removed, and the rats were placed in the pool at the farthest starting positions from the original platform location and allowed to swim for 60 s. The motion trail was recorded and analyzed using the JLBehv-M program (Jiliang) to determine the percentage of time spent in the target quadrant and the exact number of crossings over the former platform location.

**Table 1:** Modified neurological severity score.

**Task**	**Description**	**Success/Failure**
Limb hemiplegia	Any limb hemiplegia	0/1
Walk straightly	Walk actively, harmoniously and in a straight line	0/1
Tail position	Tail position is up	0/1
Auricle reflex	Shaking head when contacting the external auditory canal	0/1
Startle reflex	Motor response to loud noise	0/1
Circle exit	Able to find the exit from a large round container in 2 min	0/1
Round stick balancing	Able to balance on a 5 mm round bar for 10 s	0/1
Negative polar reflex	Able to turn back when facing down on a sloping platform	0/1
Beam walk: 1.5 cm	Slide off the beam no more than 2 times	0/1
Beam walk: 1.0 cm	Slide off the beam no more than 2 times	0/1
Beam walk: 0.8 cm	Slide off the beam no more than 2 times	0/1
Maximal score	/	11

### Data Analysis

Data are presented as the means ± SEM and were analyzed using GraphPad Prism Software. Normal values were compared using a *t*-test or one-way ANOVA if they passed the normality test (D'Agostino–Pearson omnibus normality test). The Mann–Whitney test and Kruskal–Wallis test were used for non-normally distributed data. A *p* < 0.05 was considered statistically significant.

## Results

### Sequential Pathological Changes and Myelin Deficits in the Immature Rat HI Brain Injury Model

**Figure 1:**
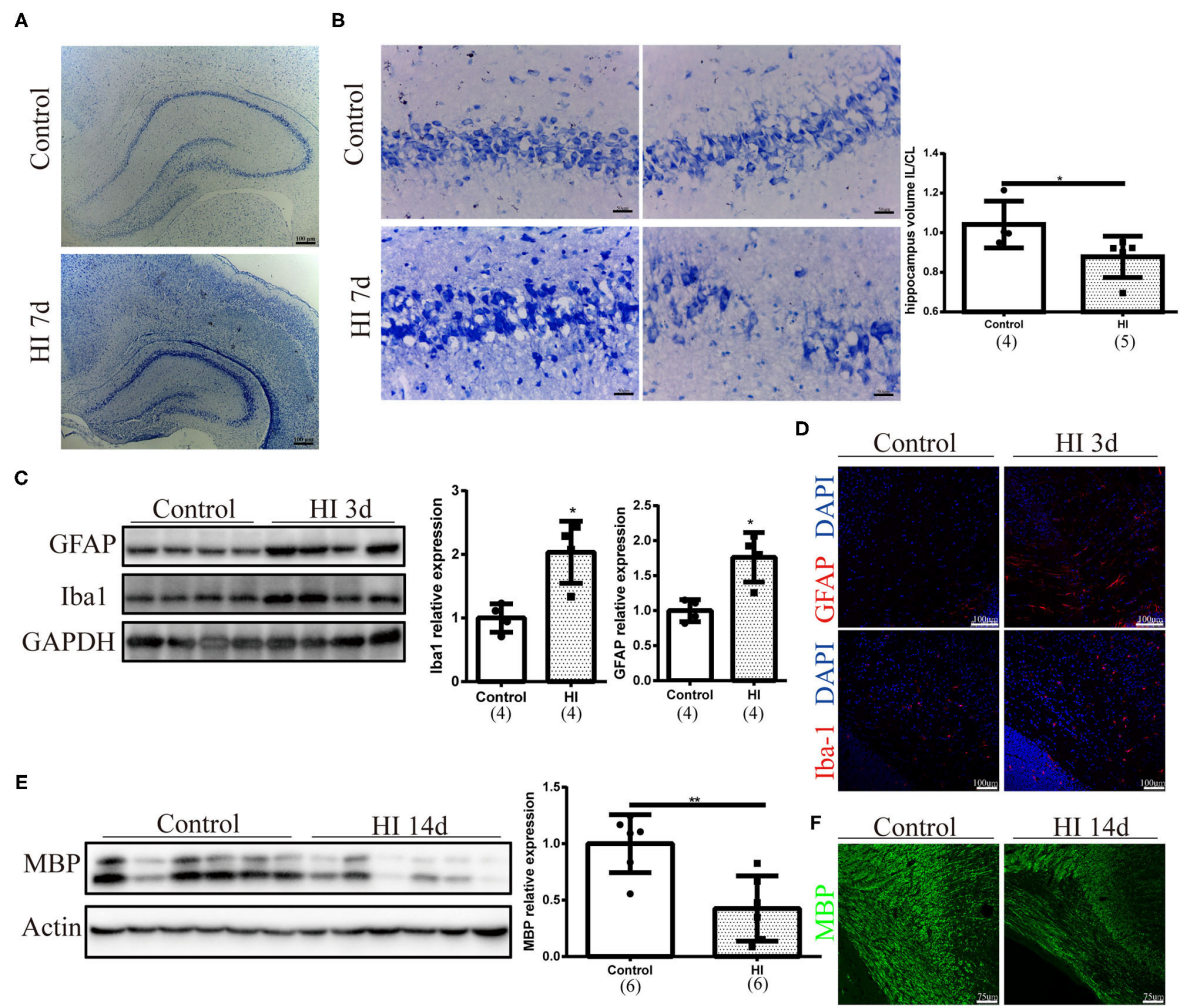
Sequential pathological changes and myelin deficits observed in the immature rat HI brain injury model. **(A)** Representative images of Nissl staining at 7 days after HI injury (HI 7 d). Scale bar: 100 μm. **(B)** Representative images and quantification of the volume ratio of hippocampus from the injured ipsilateral (IL) hemisphere to the contralateral (CL) hemisphere at HI 7 d. *N* = 4–5 animals per group. Scale bar: 50 μm. **(C)** Immunoblots and quantification of GFAP and Iba-1 levels at 3 days after injury (HI 3 d). *N* = 4 animals per group. **(D)** Images of immunofluorescence staining for GFAP and Iba-1 in the right hemisphere at 3 days post injury. Scale bar: 100 μm. **(E)** Immunoblots and quantification of myelin basic protein (MBP) levels at 14 days post injury (HI 14 d). *N* = 6 animals per group. **(F)** Images of immunofluorescence staining for MBP at HI 14 d. Scale bar: 75 μm. Values are presented as means ± SEM. *p < 0.05 and **p < 0.01.

We first performed Nissl staining to describe tissue injury at 7 days after HI injury and observed a smaller hippocampus on the ipsilateral side of HI injury (Figure 1A). Neurons in the hippocampus were arranged loosely on the ipsilateral side of HI injury and the quantification of the volume ratio of hippocampus from the injured ipsilateral (IL) hemisphere to the contralateral (CL) was decreased in the HI group (*p* = 0.032). (Figure 1B). A significant increase in GFAP (*p* = 0.029) and Iba-1 (*p* = 0.029) expression was observed in the ipsilateral side of the HI injury at 3 days after injury using Western blotting (Figure 1C). In addition, the numbers of GFAP (+) and Iba-1 (+) cells increased on the right side 3 days post injury, as determined using immunofluorescence staining (Figure 1D). Western blotting and immunofluorescence revealed a significant decrease in the expression of MBP (*p* = 0.008) on the ipsilateral side of the HI injury at 14 days post injury (Figures 1E,F).

### p-Src Levels in the Immature Rat HI Brain Injury Model

**Figure 2:**
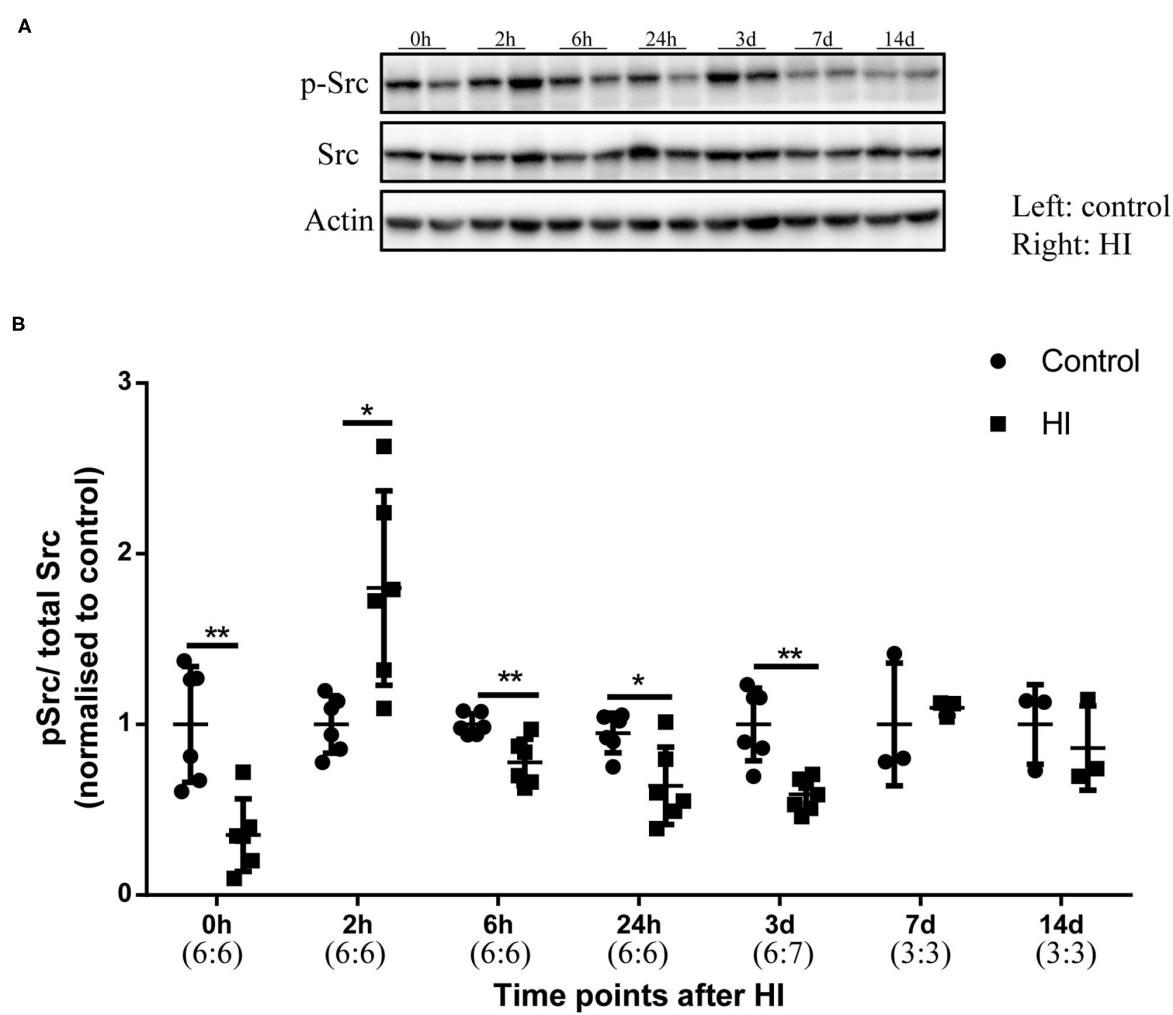
The levels of phospho-Src (p-Src) in the immature rat HI brain injury model. **(A)** Immunoblots showing the levels of p-Src and Src at different time points after HI injury. Left hemisphere, control; Right hemisphere, HI. **(B)** Quantification of the levels of p-Src and Src at different time points. Actin was used as the loading control. *N* = 3–7 animals per group. *p < 0.05 and **p < 0.01.

We performed Western blotting to investigate phospho-Src (p-Src) (Tyr416) levels from 0 h to 14 days after HI injury (Figure 2A). In Figure 2B, p-Src levels first decreased at 0 h (*p* = 0.008) after injury and increased at 2 h (*p* = 0.015) after injury. The relative p-Src levels exhibited a decreasing trend at 6 h (*p* = 0.009), 24 h (*p* = 0.026), and 3 days (*p* = 0.002). Between 7 and 14 days after injury, the relative p-Src levels returned to the control levels (*p* > 0.05).

### Activated NR2B (Tyr1472) Expression and Association of NR2B With Src Kinases in the Immature Rat HI Brain Injury Model

**Figure 3:**
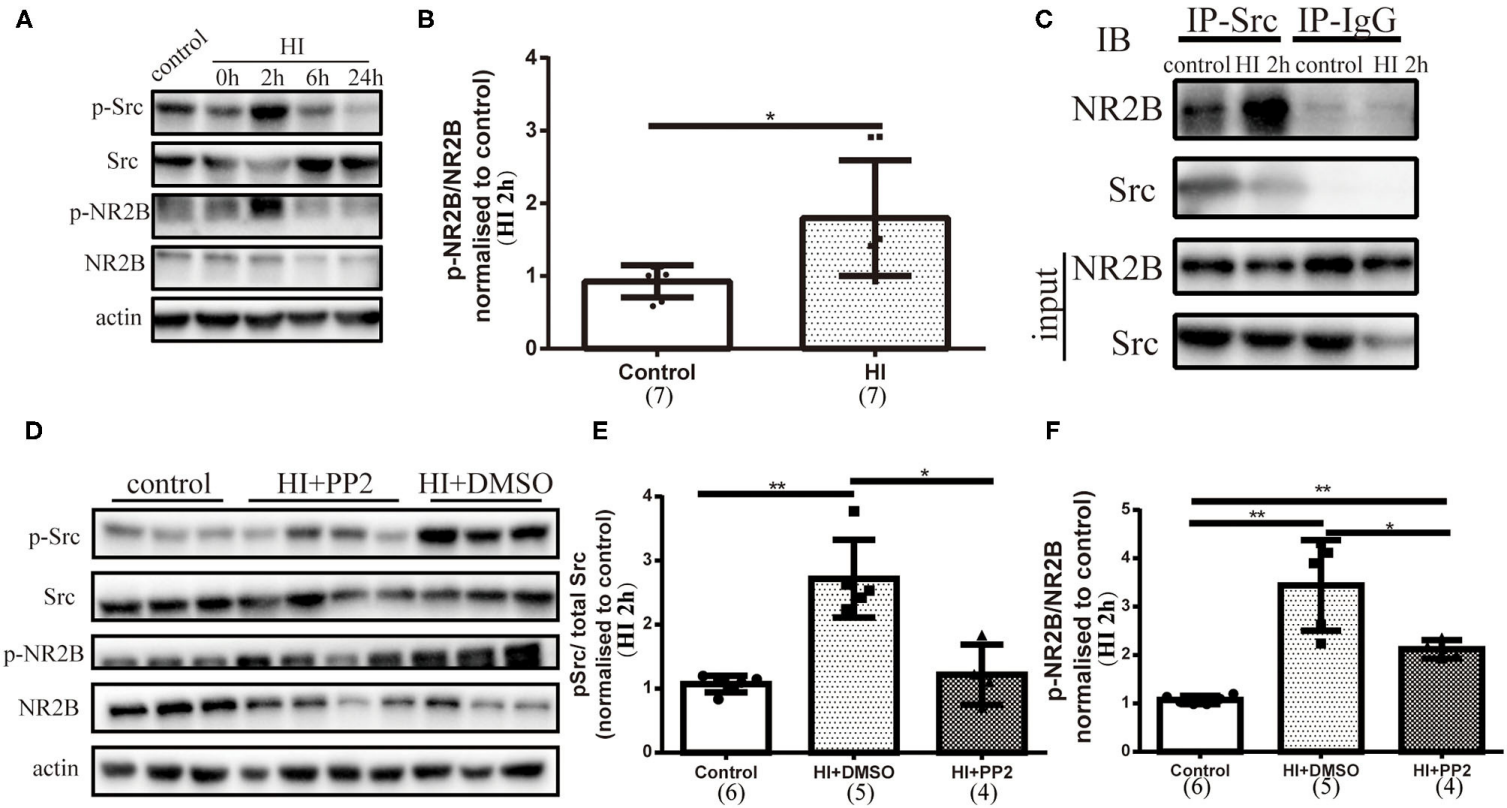
Activated NR2B (Tyr1472) expression, the enhanced interaction of NR2B with Src, and changes in the levels of p-Src and p-NR2B after the administration of 4-amino-5-(4-chlorophenyl)-7-(*t*-butyl) pyrazolo [3,4-*d*] pyrimidine (PP2) to the immature rat HI brain injury model. **(A)** Immunoblots showing the levels of multiple proteins. Primary antibodies are listed on the left. **(B)** Quantification of NR2B (Tyr1472) (p-NR2B) and NR2B levels at 2 h post injury (HI 2 h). *N* = 7 animals per group. **(C)** Coimmunoprecipitation was conducted using right hemisphere homogenates with the Src antibody or rabbit IgG as a control. Immune complexes were detected using immunoblotting (IB) with anti-Src and anti-NR2B antibodies. **(D)** Immunoblots showing the levels of multiple proteins. Primary antibodies are listed on the left. **(E)** Quantification of the levels of p-Src and Src at HI 2 h. *N* = 4–6 animals per group. **(F)** Quantification of the levels of p-NR2B and NR2B at HI 2 h. *N* = 4–6 animals per group. *p < 0.05 and **p < 0.01.

We assessed the phosphorylation of NMDARs (2B, Y1472) after HI injury by collecting tissues from the right hemisphere of rats in the control and HI groups at different time points. Along with the increased phosphorylation of Src observed at 2 h after injury, the phosphorylation of NR2B (Y1472) (p-NR2B) was increased (*p* = 0.011) (Figures 3A,B). We detected the interaction of NR2B and Src by performing immunoprecipitation and found an increased interaction of NR2B and Src (Figure 3C). Then, we performed an intraperitoneal injection of PP2 or DMSO at 30 min after HI injury and collected brain sections at 2 h post injury to investigate whether the inhibitor would alter the levels of p-Src and p-NR2B. Decreased levels of p-Src (*p* < 0.05) and p-NR2B (*p* < 0.05) were shown after HI+PP2 treatment (Figures 3D–F).

### PP2 Ameliorated Pathological Changes and Myelin Deficits in the Immature Rat HI Brain Injury Model

**Figure 4:**
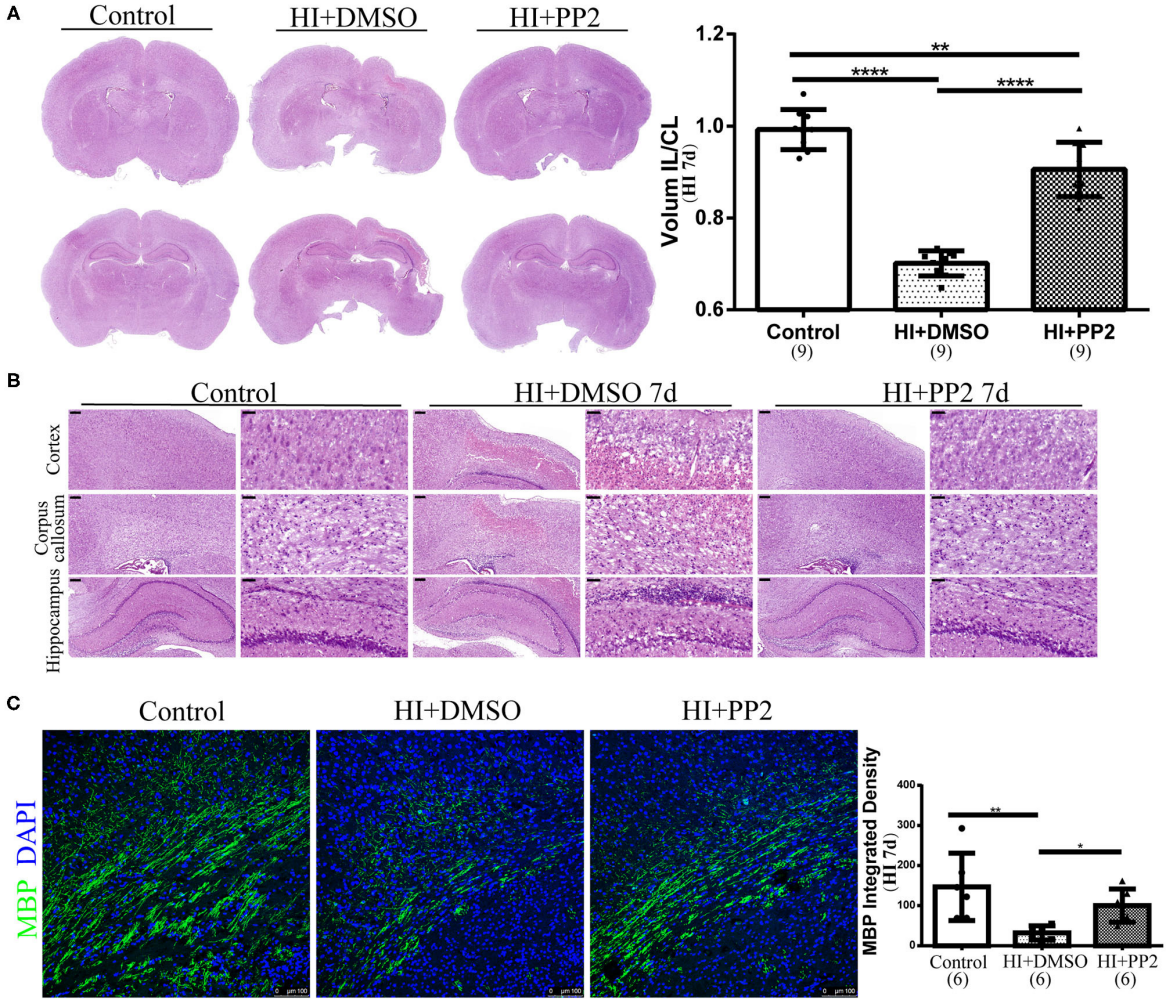
Amelioration of pathological changes and myelin deficits in the immature rat HI brain injury model after the PP2 intervention. **(A)** Representative images of H-E staining and quantification of the volume ratio of the injured IL hemisphere to the CL hemisphere at HI 7 d. *N* = 9 animals per group. **(B)** Representative images of the cortex, corpus callosum, and hippocampus in the right hemisphere of sections from control, HI+DMSO, and HI+PP2 groups. Scale bars: 200 μm (left panel) and 50 μm (right panel). **(C)** Images of immunofluorescence staining for quantification of MBP. Scale bar: 100 μm. *N* = 6 animals per group. *p < 0.05, **p < 0.01, and ****p < 0.0001.

We have already observed changes in the levels of p-Src after HI injury. Then, we used the SFK-selective inhibitor PP2 to investigate whether the inhibition of increased levels of p-Src would exert neuroprotective effects. We collected brain sections at 7 days after injury. Using H-E staining, we found that tissue loss on the ipsilateral side of the HI injury was significantly improved after the PP2 intervention (*p* < 0.0001) (Figure 4A). PP2 also decreased karyopyknosis and karyorrhexis in the cortex, increased the cell density and decreased vacuoles in the corpus callosum, and improved the loose arrangement of cells in the hippocampus on the ipsilateral side of HI injury (Figure 4B). In contrast, MBP expression was maintained at levels in comparable with controls after the PP2 intervention (*p* < 0.05) (Figure 4C).

### PP2 Intervention Improved Performance Neurological Behavioral Tests

**Figure 5:**
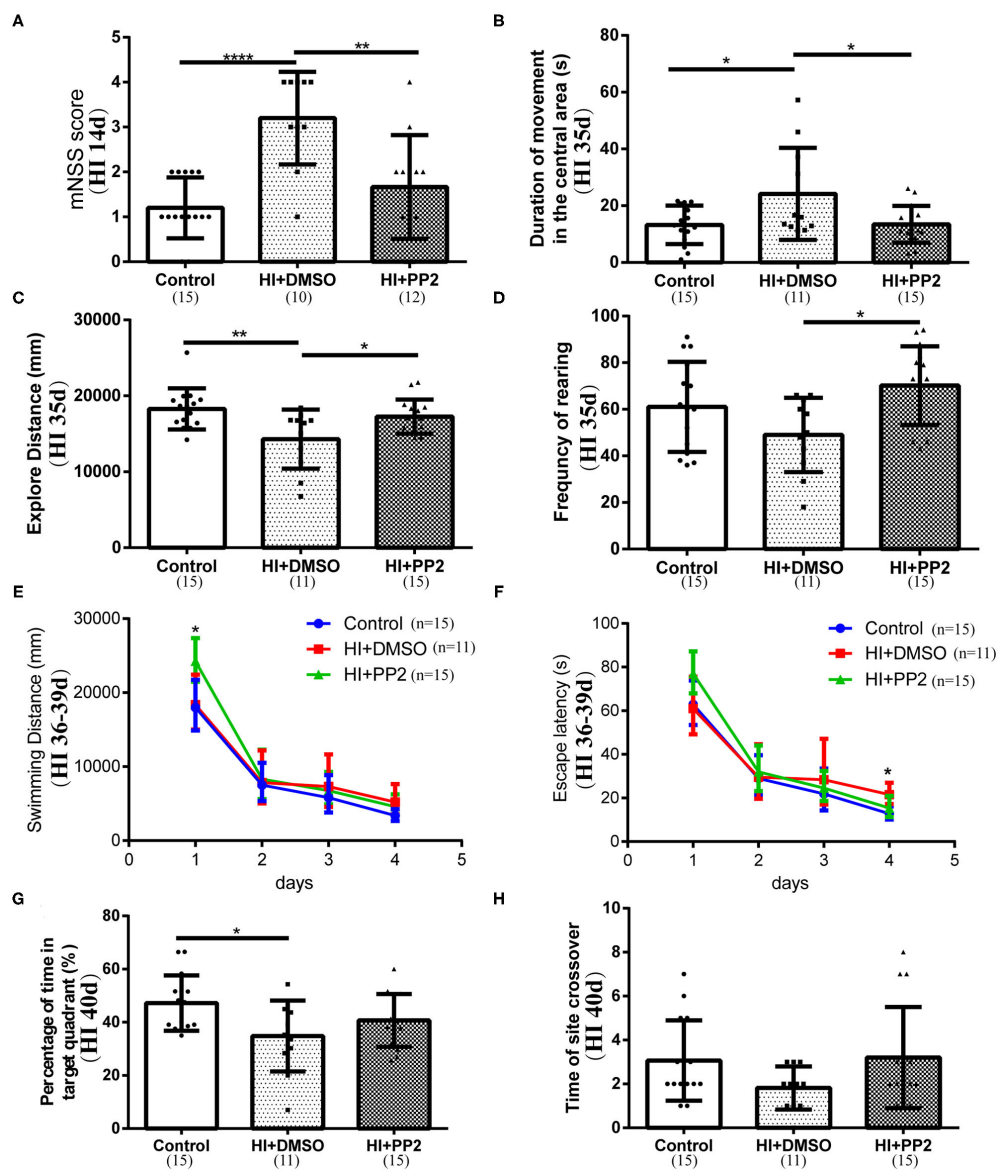
Improved performance of immature rats with HI-induced brain injury on neurological behavioral tests after the PP2 intervention. **(A)** Results of the mNSS at HI 14 d. **(B)** The duration of movements in the central area (s) of the open field test at 35 days post injury (HI 35 d). **(C)** Distance traveled (mm) in the open field test at HI 35 d. **(D)** The frequency of rearing in the open field test at HI 35 d. **(E)** Swimming distance (mm) in the Morris water maze from 36 to 39 days after injury (HI 36–39 d). **(F)** Escape latency (s) in the Morris water maze at HI 36–39 d. **(G)** Percentage of time spent in the target quadrant (%) of the Morris water maze at 40 days post injury (HI 40 d). **(H)** Number of crossings over the previous platform site in the Morris water maze at HI 40 d. *N* = 10–15 animals per group. *p < 0.05, **p < 0.01, and ****p < 0.0001.

We conducted several behavioral tests to determine whether the inhibition of increased levels of p-Src would improve neurological functions. First, we performed the mNSS test at 14 days after HI injury to evaluate motor, balance, and reflex functions. Higher scores are related to lower neurological functions. Rats in the HI+DMSO group (3.20 ± 1.03) showed higher mNSS scores than rats in the control group (1.20 ± 0.68) (compared with the control group, *p* < 0.0001), and the PP2 intervention (1.67 ± 1.15) has improved the mNSS scores (compared with the control group, *p* > 0.05; compared with the HI+DMSO group, *p* < 0.01) (Figure 5A), which reflected the improved motor, balance, and reflex functions following PP2 administration after HI. Then, we performed an open field test at 35 days after injury. The duration of movements in the central area, total walking distance, and frequency of rearing was measured to partly reflect anxiety, explore distance, and frequency of exploratory behaviors, respectively, in this test. Compared with the control group (13.26 ± 6.75 s), the duration of movements in the central area was significantly increased in the HI+DMSO group (24.16 ± 16.18 s) (compared with the control group, *p* < 0.05), which was decreased in the HI+PP2 group (13.46 ± 6.53 s) (compared with the HI+DMSO group, *p* < 0.05) (Figure 5B). Compared with the control group (18,275 ± 2,702 mm), a significant decrease in the explore distance by the HI+DMSO group was observed (14,294 ± 3,882 mm) (compared with the control group, *p* < 0.01), and the PP2 intervention (17,261 ± 2,253 mm) increased the distance traveled (compared with the HI+DMSO group, *p* < 0.05) (Figure 5C). The PP2 intervention (70.20 ± 16.87) increased the frequency of rearing compared with the HI+DMSO group (49.00 ± 15.96) (*p* < 0.05) (Figure 5D). These results of the open field test indicated that the PP2 intervention may increase the explore distance and frequency of exploratory behaviors in HI-injured rats and may decrease the duration of movements in the central area after injury, which was one of the anxiety-like behaviors. Finally, we performed the Morris water maze test at 36–40 days after HI injury. The swimming distance and time were measured from 36 to 39 days after injury, and the mean values of data collected each day were used in the analysis to evaluate the learning ability. The swimming time/escape latency and distance traveled by each group decreased with increasing training time. On the first day of training, the swimming distance was different between groups, and the HI+PP2 group exhibited a longer swimming distance (*F* = 3.68, *p* = 0.035) (Figure 5E). On the fourth day of training, the escape latency of the HI+DMSO group was longer than that of the control group (*p* < 0.05), and there was no difference between the groups HI+PP2 and HI+DMSO (Figure 5F). On the last day of the Morris water maze test, the percentage of time spent in the target quadrant and the number of crossings over the former platform site were measured to examine the spatial cognition of rats. Compared with the control group (47.25 ± 10.39 s), the HI+DMSO group spent less time in the target quadrant (34.82 ± 13.32 s) (*p* < 0.05) (Figure 5G). No difference was observed between the HI+DMSO and HI+PP2 groups (40.71 ± 9.92) (*p* > 0.05). No difference in the number of crossings over the previous platform location was observed among the three groups (*p* > 0.05) (Figure 5H).

## Discussion

In this study, we discovered changes in the phosphorylation of SFKs over time after injury, the relationship between SFKs and NMDARs, and the neuroprotective effects of PP2 administration.

The HI is one of the initial factors leading to brain injury and causes a cascade of biochemical reactions in the immature brain (Fleiss and Gressens, 2012). The phosphorylation of SFKs and NMDARs was increased at 2 h post injury. The increased phosphorylation level and interaction of SFKs and NMDARs may be a risk factor for brain injury. One study showed that brain injury was exacerbated by Fyn overexpression in a mouse HI model (Knox et al., 2013). The downstream mechanism may be related to the increased entry of Ca^2+^ via upregulated NMDARs, which may participate in intracellular signaling cascades and lead to cell death. *In vitro*, activated SFKs were related to the activation of endoplasmic reticulum inositol 1,4,5-trisphosphate receptors and increased cytosolic Ca^2+^ signaling after hypoxia-induced injury (Socodato et al., 2018). An *in vitro* study revealed that microglia activated Src and increased cytosolic Ca^2+^ signaling after hypoxia, resulting in glutamate release (Socodato et al., 2018).

According to many studies, SFKs regulate the function of NMDARs (Yu et al., 1997; Salter and Kalia, 2004). During normal development, SFK activity and NMDAR function exert some effects on regulating synaptic plasticity and metaplasticity (Iqbal Hossain et al., 2015). A study by Cao and Yao showed that physiological NMDAR signaling regulates OL differentiation and myelination through SFK-dependent signaling (Cao and Yao, 2013). Another study showed a relationship between schizophrenia susceptibility and decreased Src signaling and NMDAR responses (Pitcher et al., 2011). However, in this study, the phosphorylation of SFKs was decreased for a long time after injury (from 6 h to 3 days after injury), which may result in insufficient function. One study was designed in which neurons were treated with SFK inhibitors, and the authors discovered the treatment-induced cell death (Iqbal Hossain et al., 2015), suggesting that the catalytic activity of SFKs is critical for neuronal survival and a decrease observed after HI injury may lead to cell death and developmental disorders.

Many studies have indicated that the interaction of SFKs and NMDARs after HI injury may induce oxidative stress and excitotoxicity, which play important roles in HI brain injury. The early administration of treatment for HI brain injury may save more neural cells. The administration of the SFK-selective inhibitor PP2 at 0.5 h after injury exerted a neuroprotective effect in this study. PP2 administration improved the motor, balance, and reflex functions and increased the distance traveled and frequency of exploratory behaviors of HI rats. In an HI model constructed using neonatal piglets, intravenous injection of PP2 reduced the cytoplasmic levels of preapoptotic proteins and apoptosis, ameliorated neuropathological changes, and reduced HI-induced caspase-3 activity, possibly reducing nerve cell death (Kratimenos et al., 2017). In another study using a neonatal piglet HI model, hypothermia and PP2 administration decreased apoptosis and attenuated neuropathology after HI (Kratimenos et al., 2018). Systemic administration of PP2 reduces hippocampal neuron loss and spatial memory impairment after intraventricular hemorrhage in adult rat models (Liu et al., 2017). PP2 administration potentially represents a promising therapeutic approach for HI brain injury.

Several studies have evaluated the downstream and upstream mechanisms of SFK activation. Fyn-dependent signaling promotes myelination and regulates neural development (Wake et al., 2011). BDNF regulates myelin through Fyn activation, resulting in phosphorylation of OL-expressed Tyrosine kinase B (TrkB) receptors and ERK1/2 (Peckham et al., 2016). In one study, GABA receptor agonists promoted the activation of SFKs, suggesting that SFKs may be involved in the effect of GABA signaling on OL differentiation (Serrano-Regal et al., 2020). High concentrations of ATP increase the number of migratory OPCs *in vitro*, while 2,3-*O*-(4 benzoylbenzoyl) adenosine 5-triphosphate (P2X7 receptor agonist) exerts a stronger promoting effect than ATP, potentially through the activation of Fyn (Feng et al., 2015). Recently, a high level of phospho-c-Src was reported to be critical for neural stem cell differentiation into OPCs *in vitro* and was related to the phosphorylation of Akt and ERK1/2 (Wang et al., 2018). These mechanisms provide some suggestions for future studies, evaluating the mechanisms underlying the decreased levels of p-Src observed after HI injury in this study.

The HI injury is a factor initiating brain injury, exerting adverse effects on preterm infants. This study has proven that the levels of p-Src change over time after HI injury and that the administration of PP2 after injury may exert neuroprotective effects. However, in a neonatal mouse HI model, estrogen receptor α (ER-α) was associated with SFK activation, which in turn increased TrkB phosphorylation and reduced apoptosis, suggesting that the specific induction of ER-α expression in females induces TrkB activation through SFKs and thus plays a neuroprotective role (Cikla et al., 2016). As shown in this study, SFKs play multiple roles in the central nervous system, and inhibition of multiple SFKs after injury may produce deleterious outcomes. For HI brain injury, especially in preterm infants, treatments targeting SFKs require further exploration.

## Data Availability Statement

The original contributions presented in the study are included in the article/supplementary material, further inquiries can be directed to the corresponding author/s.

## Ethics Statement

The animal study was reviewed and approved by Children's hospital of Fudan University.

## Author Contributions

HQ and LW conceived this study. HQ, TQ, TW, and TG established the HI model and performed the experiments. HQ and LW drafted this manuscript. QX and LW reviewed and edited this manuscript. All authors contributed to this article and approved the submitted version.

## Funding

This work was supported by the National Key Research and Development Program of China Stem Cell and Translational Research (Grant No. 2017YFA0104200).

## Conflict of Interest

The authors declare that the research was conducted in the absence of any commercial or financial relationships that could be construed as a potential conflict of interest.

## Publisher's Note

All claims expressed in this article are solely those of the authors and do not necessarily represent those of their affiliated organizations, or those of the publisher, the editors and the reviewers. Any product that may be evaluated in this article, or claim that may be made by its manufacturer, is not guaranteed or endorsed by the publisher.
